# Sensory profiles in women with neuropathic pain after breast cancer surgery

**DOI:** 10.1007/s10549-020-05681-8

**Published:** 2020-05-27

**Authors:** L. Mustonen, J. Vollert, A. S. C. Rice, E. Kalso, H. Harno

**Affiliations:** 1 Division of Pain Medicine, Department of Anesthesiology, Intensive Care and Pain Medicine, Pain Clinic, University of Helsinki and Helsinki University Hospital, P.O. Box 140, 00029 HUS Helsinki, Finland; 2 Neurocenter, Neurology, University of Helsinki and Department of Neurology, Helsinki University Hospital, Helsinki, Finland; 3 Pain Research, Department of Surgery and Cancer, Imperial College London, London, UK; 4 Neurophysiology, Center of Biomedicine and Medical Technology Mannheim CBTM, Medical Faculty Mannheim, Ruprecht-Karls-University, Heidelberg, Germany

**Keywords:** Post-surgical pain, Neuropathic pain, Quantitative sensory testing, Sensory mapping

## Abstract

**Purpose:**

We performed a detailed analysis of sensory function in patients with chronic post-surgical neuropathic pain (NP) after breast cancer treatments by quantitative sensory testing (QST) with DFNS (German Research Network on Neuropathic Pain) protocol and bed side examination (BE). The nature of sensory changes in peripheral NP may reflect distinct pathophysiological backgrounds that can guide the treatment choices. NP with sensory gain (i.e., hyperesthesia, hyperalgesia, allodynia) has been shown to respond to Na^+^-channel blockers (e.g., oxcarbazepine).

**Methods:**

104 patients with at least “probable” NP in the surgical area were included. All patients had been treated for breast cancer 4–9 years ago and the handling of the intercostobrachial nerve (ICBN) was verified by the surgeon. QST was conducted at the site of NP in the surgical or nearby area and the corresponding contralateral area. BE covered the upper body and sensory abnormalities were marked on body maps and digitalized for area calculation. The outcomes of BE and QST were compared to assess the value of QST in the sensory examination of this patient group.

**Results:**

Loss of function in both small and large fibers was a prominent feature in QST in the area of post-surgical NP. QST profiles did not differ between spared and resected ICBN. In BE, hypoesthesia on multiple modalities was highly prevalent. The presence of sensory gain in BE was associated with more intense pain.

**Conclusions:**

Extensive sensory loss is characteristic for chronic post-surgical NP several years after treatment for breast cancer. These patients are unlikely to respond to Na^+^-channel blockers.

## Introduction

Chronic post-surgical neuropathic pain (NP, ICD 11 codes MG30.51 Chronic neuropathic pain after peripheral nerve injury and MG30.11 Chronic post cancer treatment pain) is common after breast cancer surgery with an estimated prevalence of 14–31% [1, 2]. This condition may persist for years [3, 4]. Surgical trauma to the intercostobrachial nerve (ICBN) is postulated as a major cause of both sensory impairment and NP [5, 6] although injuries to other nerves (e.g., intercostal nerves) may also contribute to post-surgical NP [7].

The features of sensory dysfunctions in peripheral NP may reflect distinct pathophysiological backgrounds with different responses to medical treatments [8–11]. Clinical bedside examination (BE) of sensory function is the essential first step of demonstrating “the presence of a lesion or disease of the somatosensory system” in the diagnostic workup of NP [12]. Quantitative sensory testing (QST) allows for more detailed and quantified assessment of sensory function. Recent studies on standardized QST protocol developed by the DFNS (German Research Network on Neuropathic Pain) have provided new data of sensory profiles across NP etiologies [13, 14].

After breast cancer surgery, sensory loss in QST is a prominent finding, although some patients present with sensory gain [15–17]. However, QST studies in this patient group are scarce, especially with long follow-up after surgery. Previous studies using DFNS QST for post-surgical NP in general have included both surgical and traumatic etiologies [18–20] or have had a small cohort [21]. Patients with orthognathic surgery showed postoperative sensory loss, but recovered well in three month follow-up [21]. To the best of our knowledge, no studies using the DFNS QST protocol have been conducted in the breast cancer surgery patient group before.

Our study had two aims. Firstly, we wanted to perform a detailed sensory characterization of patients with chronic post-surgical NP several years after treatments. We assessed QST by the DFNS protocol and performed sensory mapping by detailed BE. Secondly, we wanted to assess what added value QST might offer to the clinical BE in this patient group. For this, we compared the outcomes of QST and BE. With these measures, we aimed to improve understanding and management of post-surgical pain in breast cancer survivors.

## Patients and methods

### Patients

All patients were recruited from a previous cohort of 1000 women operated for breast cancer at the Helsinki University Hospital during 2006–2010 [22]. 402 of them participated in a new study (project acronym NeuroPain) on post-surgical NP during 2014–2016. Patients with a surgeon-verified ICBN resection and patients who had reported post-surgical pain in the annual follow-up questionnaires were invited. Patients under 75 years were included.

We graded the diagnosis of NP according to the revised diagnostic criteria of NP [23]. For the current study, we included patients who reached the diagnostic level of “probable” NP, i.e., patients who had relevant pain history in the neuroanatomically plausible area and at least one abnormal clinical sensory finding at the site of pain in BE [4, 23]. We excluded patients with breast reconstruction, bilateral surgery, bilateral pain, or other reason that may affect sensory function (e.g., previous varicella zoster eruption), and psychiatric/cognitive reasons.

The research-visit included a structured neurological examination with pain assessments and BE for sensory function of the upper body. The examining neurologist (HH) was blinded to the ICBN resection status. Patients gave self-reports of their current medication. The eligible patients were invited for the QST. Two DFNS-certified nurses performed QST. The study was registered in the ClinicalTrials.gov (NCT02487524).

### Cancer treatments

The data of cancer treatments were available from the previous study [22]. The surgical procedure was either mastectomy or breast conserving resection (BCR) accompanied with either sentinel lymph node biopsy (SLNB) or axillary lymph node dissection (ALND). The operating surgeon registered the ICBN handling on a 4-point categorical scale (spared, partially or totally resected, not visualized). The oncological treatments were administered according to national treatment protocols. Chemotherapy regimen consisted of a combination of docetaxel and CEF (cyclophosphamide, ebirubicine, and 5-fluorouracil). Endocrine therapy consisted of tamoxifen (premenopausal women) or aromatase inhibitor (postmenopausal women).

### Pain assessments

We use the term “surgical area” for the operated breast and the area that is neuroanatomically plausible for the ICBN lesion (lateral breast, upper chest wall, axilla, medial upper arm) [24].

For self-reported pain we used Brief Pain Inventory (BPI) [25] to rate the worst pain past week in the surgical area with Numerical Rating Scale, NRS 0–10, and marked the pain localization on a body map. For evoked pain at BE, we asked patients to rate their pain by NRS 0–10 and the examiner marked the pain site on a body map.

### Quantitative sensory testing

QST was performed by using the DFNS protocol and the equipment approved by DFNS [26]. Two nurses were trained in a DFNS-certified laboratory (September 2015, Neurophysiology, University Medicine Mannheim, Heidelberg University, Mannheim, Germany). QST was conducted at the most representative site of NP (i.e., the site with most intensive pain accompanied with sensory findings) within the surgical area and the corresponding area at the unaffected side.

The QST data for each individual patient were compared with the reference values from the DFNS normative data for the anatomically closest area of the thoracic wall [27]. The reference values are matched for age and sex. In addition, we used the unaffected contralateral area as reference when evaluating QST abnormality.

The DFNS QST studies the function of Aβ-, Aδ- and C-fibers with the following 13 tests: cold and warm detection thresholds (CDT and WDT), thermal sensory limen (TSL, ability to detect thermal changes), paradoxical heat sensations (PHS, reports of heat when the temperature is cooling), cold and heat pain thresholds (CPT and HPT), mechanical detection threshold (MDT), mechanical pain threshold and sensitivity (MPT and MPS), wind-up ratio (WUR, pain after repetitive pinprick stimulation), dynamical mechanical allodynia (DMA), pressure pain threshold (PPT), and vibration detection threshold (VDT).

VDT was performed on the clavicle for both the surgical and the corresponding unaffected side. All other tests were performed on the most representative area of NP and the respective contralateral area. Thermal tests were performed with Medoc TSA 2001-II (Ramat Yishai, Israel).

We tested the equivalence of QST and BE outcomes by comparing abnormal QST findings with sensory signs at BE. The QST finding was considered abnormal if the difference to the unaffected side exceeded 95% CI of the side-to-side difference in the normative data [27]. The outcomes were regarded equal for the following outcome pairs (BE/QST): sensory loss/abnormal loss of function; sensory gain/abnormal gain of function; no sensory abnormalities/normal function.

Based on the DFNS QST, according to the recently published algorithm [13, 14], the patients were categorized to four different sensory phenotypes, which have been demonstrated to relate to experimentally induced models of neuropathy [28].

### Bedside examination

BE covered the upper body. The following five modalities were tested: light touch (cotton tuft), dynamic allodynia (painter’s brush), static allodynia (compressing by finger), pinprick (wooden cocktail stick), cold and warm sensation (metal roller). The following sensory abnormalities were assessed for each modality: hypoesthesia (decreased sensitivity), hyperesthesia (increased sensitivity), dysesthesia (unpleasant sensation), hyperalgesia (pain evoked by pinprick), and allodynia (pain elicited by a non-painful stimulus). The unaffected side and the surrounding upper body areas, were used as reference. The examining researcher marked the areas and types of sensory abnormalities on a body map for each patient.

For data digitalization, we assessed sensory loss and sensory gain separately. In the digitalized images, we defined the area of sensory loss as that consisting of hypoesthesia in any of the tested sensory modalities. Likewise, the area of sensory gain was defined as consisting of hyperesthesia, hyperalgesia, dysesthesia, and allodynia in any of the sensory modalities. For area calculation, we used digitalized body maps. We overlapped these areas for illustration and scaled to the size of 9.0 × 15.0 cm. We report the areas of sensory abnormalities in arbitrary units (1 arbitrary unit corresponds to 1 cm^2^). We used Incscape 0.92 for area calculation and image creation.

### Statistical analyses

The values of 11 QST items (excluding PHS and DMA) were z-transformed by using the mean and standard deviation of the normative data. Z-score of zero represents the mean of the normative data. Z-scores above zero indicate gain of function and z-scores below zero indicate loss of function. Z-scores outside 95% CI of the normative data were considered abnormal.

PHS is shown as the number of occurrence (0–3) and DMA is shown as log NRS (0–100). For group comparisons, we used Student *t*-test (two groups) and ANOVA (more than two groups) for normally distributed continuous variables. For non-normally distributed continuous variables we used Mann–Whitney *U* test (two groups) and Kruskall Wallis test (more than two groups). We used *χ*^2^ test for categorical variables. Correlations were assessed with Spearman’s rho (r_S_). Linear regression analysis was used to investigate the association of the area of sensory loss and pain intensity. Logistic regression analysis was used for the association between the presence of sensory gain and pain intensity. *p* values < 0.05 were considered statistically significant.

## Results

### Patient description

**Fig. 1:**
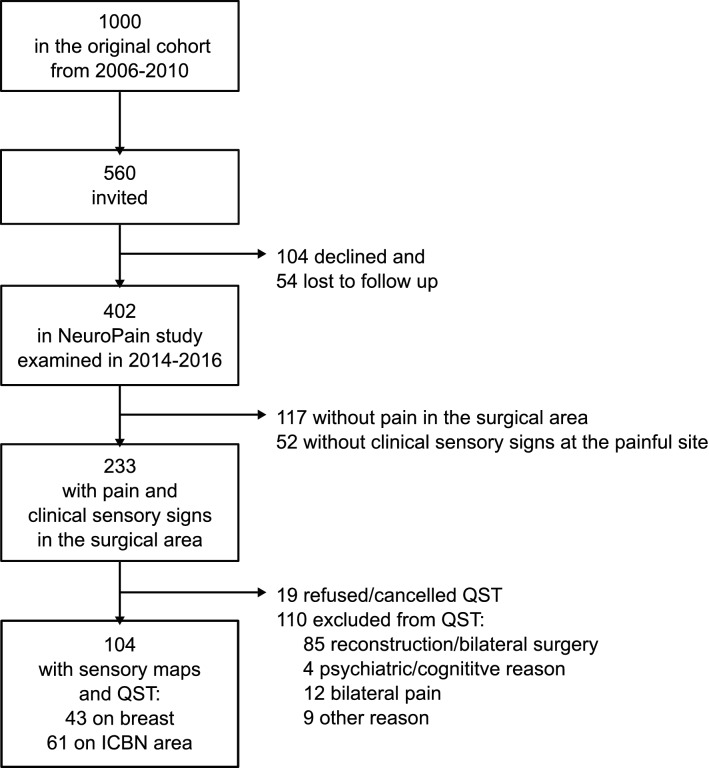
Patient flow chart

Figure 1 illustrates the patient flow. Of the 402 women operated for breast cancer and studied in the NeuroPain project, 233 patients had pain in the surgical area with associating clinical sensory signs, i.e., probable NP. 110/233 (47%) were excluded and 19/104 (18%) refused QST. Reconstruction was the most common reason for exclusion. 104 patients were examined for the current study.

Since many patients had multiple painful sites within the surgical area, the most representative area of NP was selected for QST. QST was performed on the breast for 43/104 (41%) and on the ICBN area for 61/104 (59%).

Patient characteristics and treatment-related factors are summarized in Table 1. 22/104 (21%) patients reported at least moderate (NRS 4–10) pain in the surgical area. At clinical examination, 79/101 (78%, 3 missing values) had evoked pain of at least moderate intensity.

**Table 1:** Patient description

*N* = 104	
Descriptives	Mean (SD)
Age (years)	62.3 (7.1)
Time from surgery (months)	77.6 (14.0)
Characteristics of breast cancer	*N* (%)
Histology	
Intraductal carcinoma	67 (64.4)
Intralobular carcinoma	19 (18.3)
Other	18 (17.3)
Gradus	
I	27 (26.0)
II	42 (40.4)
III	35 (33.7)
Cancer treatment	*N* (%)
Breast surgery type	
Breast conserving resection	83 (79.8)
Mastectomy	21 (20.2)
Axillary surgery type	
Sentinel lymph node biopsy	35 (33.7)
Axillary lymph node dissection	69 (66.3)
Handling of ICBN	
Spared	22 (21.2)
Partially resected	46 (44.2)
Totally resected	26 (25.0)
Not identified	10 (9.6)
Chemotherapy^a^	75 (72.1)
Docetaxel	66 (63.4)
Cyclophosphamide-ebirubicine-5-fluorouracil	72 (69.2)
Radiotherapy	94 (90.4)
Endocrine therapy	82 (78.8)
Tamoxiphen	68 (65.4)
Aromatase inhibitor	62 (59.6)

^a^One patient had received oxaliplatin for intestinal cancer

Of the patients, 8/104 (8%) reported current use of NP medication: 5/8 used amitriptyline (10–50 mg per day), 1/8 nortriptyline (75 mg per day), 2/8 pregabalin (300–375 mg per day), and 2/8 venlafaxine (75 mg per day). Other pain medication was used by 25/104 (24%) patients: 15/25 used acetaminophen, 14/25 nonsteroidal anti-inflammatory drugs (NSAID), and 6/25 mild opioids. None of the patients used strong opioids.

### Quantitative sensory testing

**Fig. 2:**
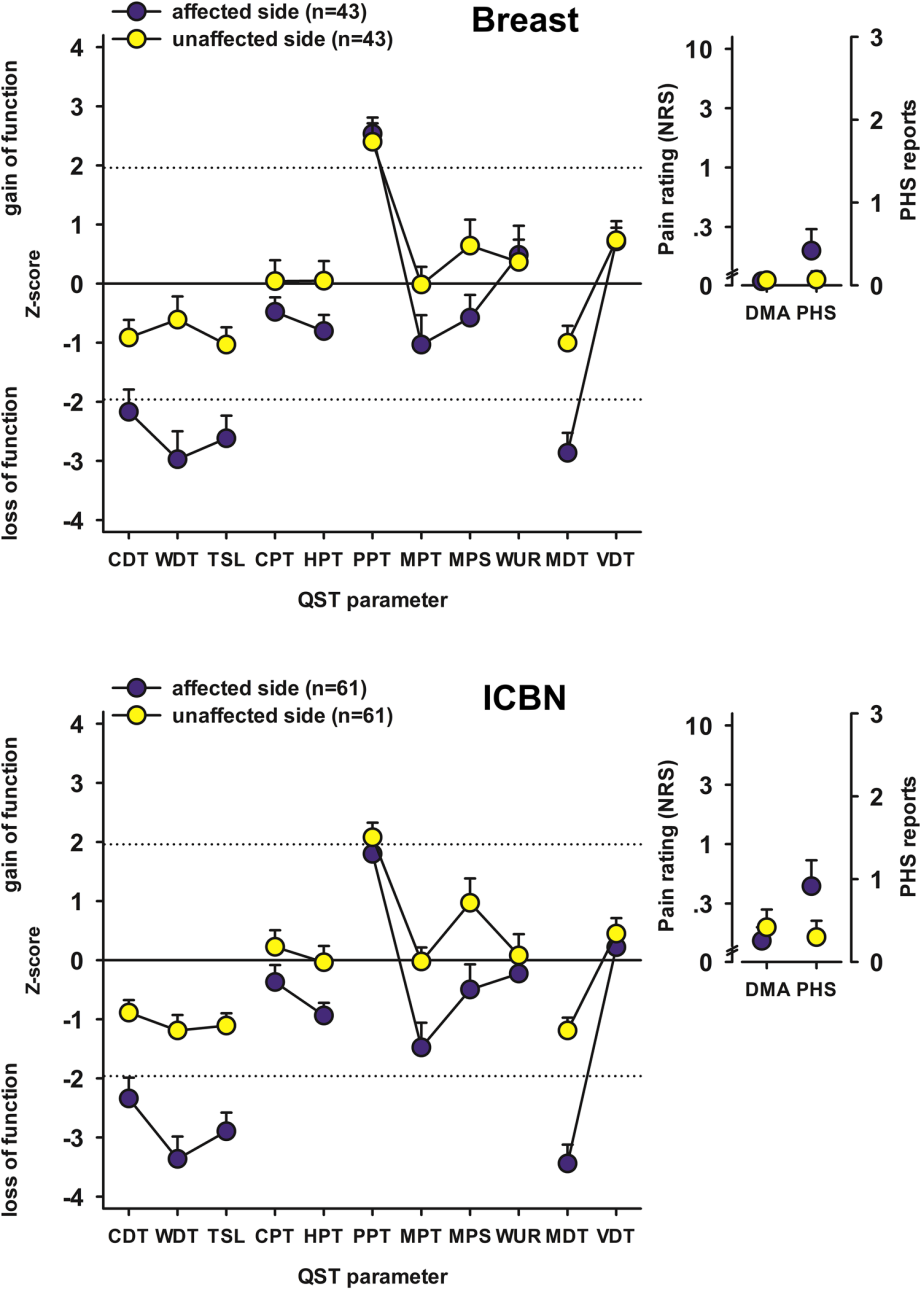
Comparison of the QST profiles on affected (surgical) and unaffected side. QST was performed on the area of breast in 43 (41%) and on the area of ICBN innervation (lateral breast, upper side of chest, axilla, upper medial arm) in 61 (59%). *CDT* cold detection threshold, *CPT* cold pain threshold, *DMA* dynamic mechanical allodynia, *HPT* heat pain threshold, *ICBN* intercostobrachial nerve, *MDT* mechanical detection threshold, *MPS* mechanical pain sensitivity, *MPT* mechanical pain threshold, *NRS* numerical rating scale, *PHS* paradoxal heat sensation, *PPT* pressure pain threshold, *QST* quantitative sensory testing, *VDT* vibration detection threshold, *WDT* warm detection threshold, *WUR* wind-up ratio

QST profiles on the breast and ICBN area are shown for the surgical (affected) and the unaffected side in Fig. 2. On the affected side, the z-scores indicated a significant (*p* < 0.001) loss of function in all items except for WUR, PPT and VDT compared with the reference data [27]. PPT and VDT showed a significant (*p* < 0.01) gain of function. On the unaffected side, the z-scores indicated a significant (*p* < 0.01) loss of function compared with the reference data for CDT, WDT, TSL, and MDT. MPS, VDT, and PPT showed a significant (*p* < 0.01) gain of function.

When comparing with the unaffected side, the z-scores on the affected side differed significantly (p < 0.05) in CDT, WDT, TSL, CPT, HPT, MDT, and MPT both on the breast and on the ICBN area (Fig. 2). MPS differed significantly on the breast and VDT on the ICBN area compared with the unaffected side. PHS was significantly more frequent in the affected side in both tested areas.

**Fig. 3:**
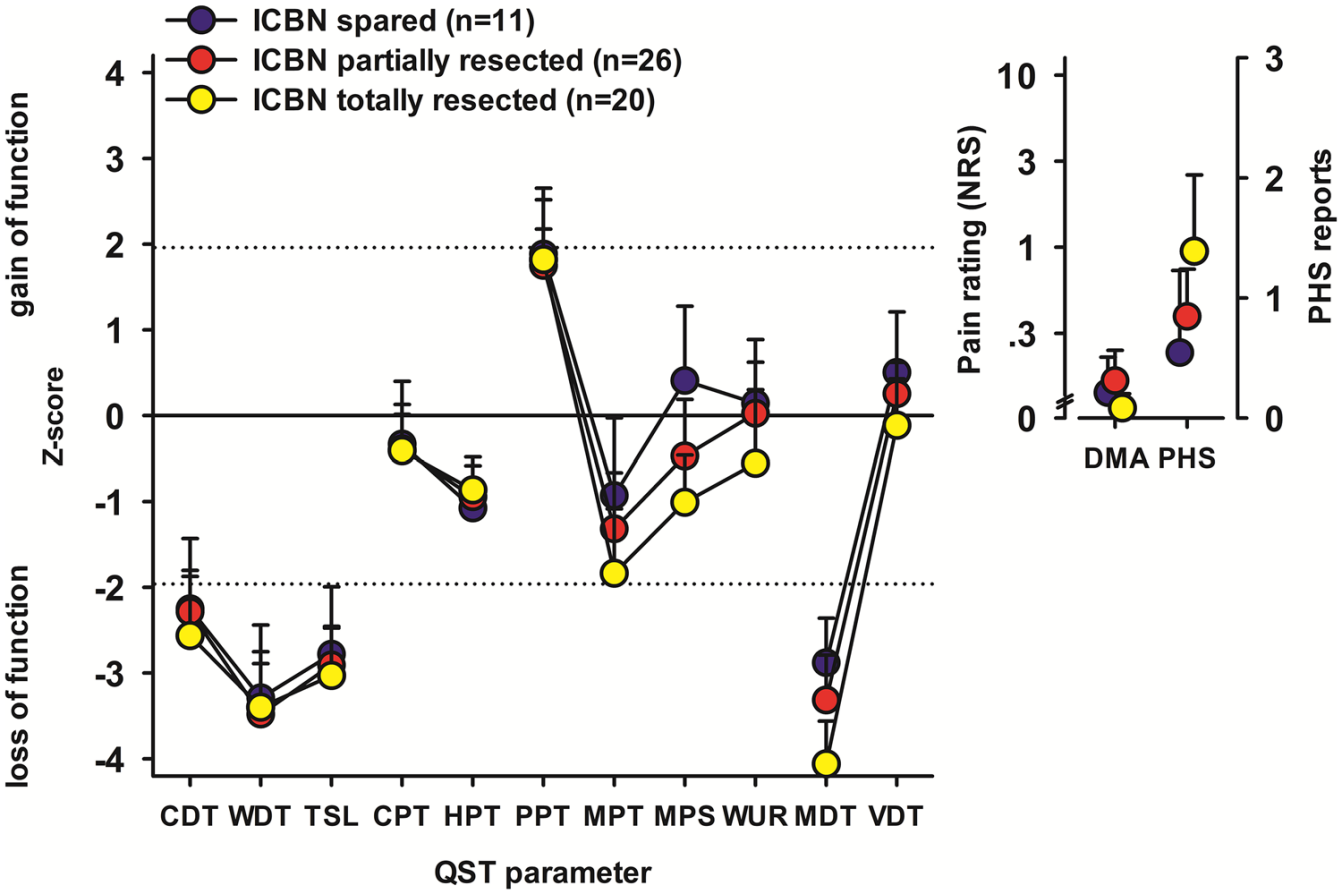
QST profiles for patients with spared (*n* = 11), partially (*n* = 26), and totally (*n* = 20) resected ICBN. *CDT* cold detection threshold, *CPT* cold pain threshold, *DMA* dynamic mechanical allodynia, *HPT* heat pain threshold, *ICBN* intercostobrachial nerve, *MDT* mechanical detection threshold, *MPS* mechanical pain sensitivity, *MPT* mechanical pain threshold, *NRS* numerical rating scale, *PHS* paradoxal heat sensation, *PPT* pressure pain threshold, *QST* quantitative sensory testing, *VDT* vibration detection threshold, *WDT* warm detection threshold, *WUR* wind-up ratio

In patients who had the QST performed on the ICBN area, we tested how the handling of ICBN may affect the sensory function. The QST profiles for patients with spared, partially, and totally resected ICBN are shown in Fig. 3. Compared with spared ICBN, patients with total resection presented with more severe loss of function in the mechanical tests for both small (MPT, MPS, WUR) and large (MDT, VDT) fiber function. However, the difference was significant (*p* = 0.031) only for MDT. Patients with totally resected ICBN reported PHS more frequently, but the difference was not significant (*p* = 0.145).

**Fig. 4:**
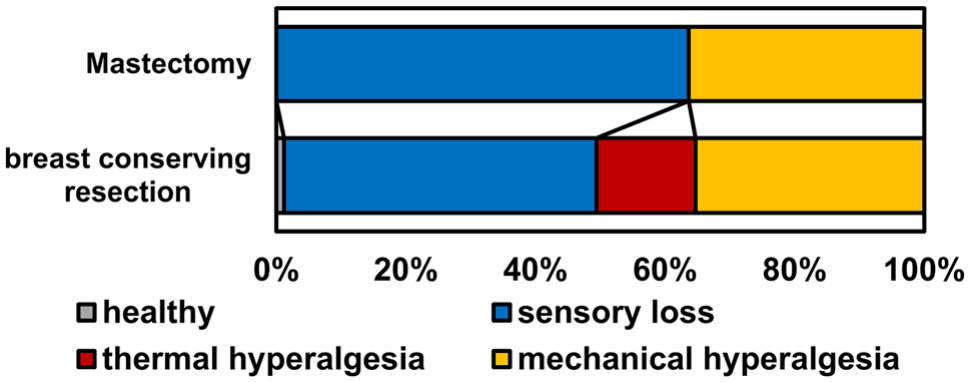
Sensory phenotypes for breast conserving resection (BCR) and mastectomy

Figure 4 presents sensory phenotypes according to the published algorithm [13, 14].”Sensory loss” was the most common phenotype (54/104, 52%) followed by “mechanical hyperalgesia” (38/104, 37%). “Thermal hyperalgesia” was present in 11/104 (11%) of patients. Sensory phenotype did not associate significantly to any of the factors listed in Table 1 or to the site of QST (data not shown). All patients with the “thermal hyperalgesia” phenotype had undergone BCR and all but one had received radiotherapy. Patients with different sensory phenotypes did not differ in terms of chemotherapy or endocrine therapy. Only one patient presented with a healthy phenotype.

The value for WUR was missing from 42/104 (40%) patients on the surgical side and from 11/104 (11%) on the unaffected side due to hyposensitivity to pinprick. Seven patients could not tolerate testing with the pressure algometer device due to high sensitivity to pressure.

### Bedside examination and sensory mapping

Sensory loss was the leading finding in BE: all patients except for two had sensory loss in at least one of the tested modalities (98%). Of these, 85/102 (83%) presented with sensory loss in all five modalities.

Sensory gain in BE was found in 38/104 (37%) patients. Of these, 11/38 (29%) had sensory gain in one modality, and 4/38 (11%) in all five modalities. Sensory gain for pinprick was most prevalent (32/38, 84%).

**Fig. 5:**
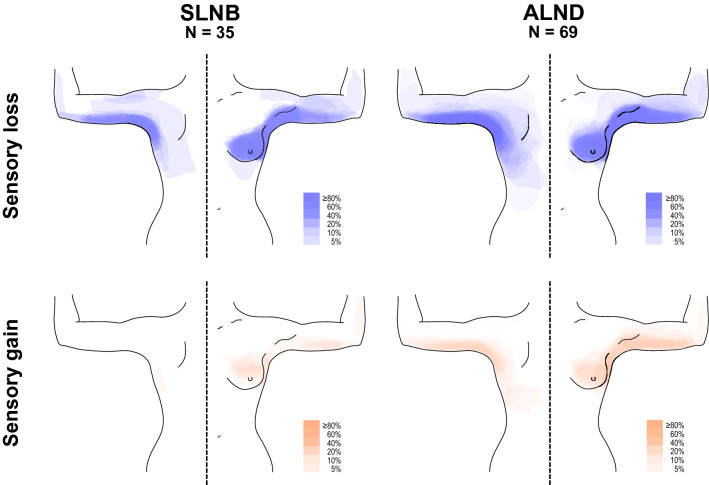
Areas of sensory abnormalities in BE on the side of surgery in patients with SLNB or ALND. Overlap of areas of sensory loss or gain in BE including light touch (cotton tuft), dynamic touch (painter’s brush), static allodynia (compression of finger), pinprick (cocktail stick), cold and warm sensation (metal roller). Sensory loss refers to hypoestesia and sensory gain refers to hyperestesia, dysestesia, or allodynia in any of these sensory modalities. For clarity, all surgeries are shown on left side. *ALND* axillary lymph node dissection, *BE* bedside examination, *ICBN* intercostobrachial nerve, *SLNB* sentinel lymph node biopsy

The overlap and magnitude of the abnormal sensory areas are illustrated in Fig. 5. In a linear regression model, including the cancer- and treatment—related factors listed in Table 1, only ALND significantly associated with a larger area of sensory loss (beta 0.336, *p* = 0.028). Thus, we presented the overlap of the areas separately for patients with SLNB (*n* = 35) and ALND (*n* = 69). For clarity, all surgeries are illustrated on the left side. Of the surgeries, 50/104 (48%) were right-sided.

9/35 (26%) of the patients with SLNB and 63/69 (91%) of the patients with ALND had a surgeon-verified ICBN resection (*p* < 0.001).

We studied the association between sensory abnormalities and pain intensity (Table 2). The intensity of self-reported pain in the surgical area (0–10 NRS) correlated with the size of the area for sensory loss. For sensory gain, we used dichotomized variable (yes/no). Patients with sensory gain reported significantly higher intensities of pain (median NRS 3/10 vs. 1/10, *p* = 0.004) even after controlling for the axillary surgery type and age compared with those without sensory gain.

**Table 2:** Associations between pain intensity and sensory sings in the surgical area

Self-reported pain (0–10 NRS)	Bivariable analyses		Multivariable adjustment^a^	
Positive sensory signs	median (IQR)	*p*-value	Beta	*p*-value
Yes	3 (1–4)	**0.004**	0.243	**0.014**
No	1 (0–3)			

	r_S_	*p*-value	Beta	*p*-value
Negative sensory sings (arb. units)	0.350	** < 0.001**	0.175	0.090
All sensory sings (arb. units)	0.365	** < 0.001**	0.183	0.081

*Arb. unit* arbitrary unit, *Beta* standardized regression coefficient, *IQR* interquartile range, *NRS* Numerical Rating Scale, *r*_*S*_ Spearman's rho

*p*-values < 0.05 are shown in bold. Since the majority of the patients did not have positive sensory sings, the variable is dichotomized for the analysis. Arb. unit refers to the measure of the area in the body maps

^a^Adjusted for the following: age and type of axillary surgery

### Comparison of the outcomes of BE and QST

BE and the corresponding QST items showed equal outcomes in 34–57% of patients (Table 3). The QST z-scores did not differ significantly in patients with sensory gain, sensory loss or normal finding in the corresponding BE test (data not shown).

**Table 3:** Frequency of equal outcomes in bedside examination and quantitative sensory testing

Clinical sensory test	Axon type	Corresponding QST item	Equal outcome in BE and QST
Static mechanical allodynia	C	PPT	33/94 (34%)
Light touch	Aβ	MDT	58/102 (57%)
Dynamic touch	Aβ	MDT	58/103 (56%)
Pinprick	Aδ	MPT	38/103 (37%)
		MPS	52/99 (53%)
Cold sensation	Aδ	CDT	49/104 (47%)
		CPT	30/102 (29%)
		TSL	57/104 (55%)
Warm sensation	C	WDT	47/104 (45%)
		HPT	36/102 (35%)
		TSL	53/104 (51%)

*BE* bedside examination, *CDT* cold detection threshold, *CPT* cold pain threshold, *HPT* heat pain threshold, *MDT* mechanical detection threshold, *MPS* mechanical pain sensitivity, *MPT* mechanical pain threshold, *PPT* pressure pain threshold, *QST* quantitative sensory testing, *TSL* thermal sensory limen

The three QST phenotypes differed very little in BE. Only sensory loss to cold sensation in BE was significantly (*p* = 0.007) less frequent in the “thermal hyperalgesia” group (4/11, 36%) compared with the “sensory loss” (44/54, 82%) and the “mechanical hyperalgesia” (29/38, 76%) groups.

## Discussion

In the DFNS QST of women with chronic post-surgical NP after breast cancer surgery, we found significant sensory loss in thermal and mechanical tests both in the affected and unaffected side when compared with the DFNS normative data. The affected side presented significant sensory loss in both thermal and mechanical tests compared with the unaffected side.

Patients with spared ICBN presented with similar sensory loss compared with resected ICBN. Of the sensory profiles, “sensory loss” was the most common (52%), followed by “mechanical hyperalgesia” (37%). Only 11% of patients, all having had BCR, presented with the “thermal hyperalgesia” profile.

In BE, sensory loss was the most prevalent (98%) finding. Sensory gain in BE was present in 37% and it was associated with more intense pain.

### Quantitative sensory testing

Few previous studies have conducted QST (not with the DFNS protocol) on the painful surgical area in patients operated for breast cancer [15, 16, 29]. They report higher thermal and tactile detection thresholds in the operated side compared with the unaffected side, in line with our results. In the long-term, the post-surgical sensory dysfunction in breast cancer survivors seems to sustain with sensory loss.

Similar to our results, previous studies also report gain of function in PPT [15, 16, 30]. However, we observed sensory gain in PPT both in the affected and unaffected side. The DFNS reference data on upper back may generate bias [27]. However, some patients were extremely sensitive even to light pressure. Moreover, widespread pressure-evoked hyperalgesia in non-surgical areas has been reported in patients operated for breast cancer [31]. Widespread sensory gain in PPT may reflect central sensitization in patients with NP after breast cancer treatments [31].

The effect of ICBN handling on chronic post-surgical pain is controversial [6]. Previously, it was reported that the risk of post-surgical pain increased if the surgeon spared ICBN [32]. However, in another recent study, resection of ICBN increased risk for NP [5].

We found a considerable sensory impairment in the ICBN area even when the nerve was spared from resection. This suggest that the patients who have NP in the ICBN innervation area have similarly impaired sensory function regardless of the surgical nerve handling. Therefore, other perioperative lesions (e.g., compression, stretching, and scar formation) to ICBN, not only mere resection, may play an important role in post-surgical NP. Our results highlight the importance of QST in further studies to better understand the effect of the type of nerve injury on post-surgical NP.

Interindividual differences in sensory profiles may reflect distinct pathophysiological backgrounds with different responses to medical treatments [13, 14, 28]. A recently published algorithm stratifies patients to three different pathological phenotypes according to the sensory profile in DFNS QST [13]. There are a few studies that support the idea of personalized NP treatments according to sensory phenotype [10, 13, 33].

Frequencies of the three QST phenotypes vary across NP etiologies [8]. In our cohort of chronic post-surgical NP patients, “sensory loss” was the most prevalent (52%) reflecting the loss of both small and large nerve fiber function. Pain may be generated by ectopic activity at the sites proximal to injury [8, 34]. 37% in this cohort belonged to the “mechanical hyperalgesia” phenotype characterized by central sensitization to mechanical stimuli and sensory loss in thermal sensation [13].

“Thermal hyperalgesia” was the most infrequent (11%) phenotype in our cohort. It is considered as a peripheral sensitization phenotype with possible response to oxcarbazepine treatment [10, 13]. It may reflect effective nerve regeneration [13], which could be hindered by radiotherapy after breast cancer surgery [35]. The high prevalence of radiotherapy in our cohort (90%) may partly explain the scarcity of “thermal hyperalgesia” phenotype. However, all patients with “thermal hyperalgesia” had undergone BCR. This is in line with a previous study reporting less severe sensory impairment in BCR compared with mastectomy [15].

The distribution of the sensory phenotypes in our cohort differs from the previously published data on patients with NP after peripheral nerve injuries reporting “thermal hyperalgesia” as the most prevalent phenotype (40%) [13]. This may suggest that post-surgical NP after breast cancer treatment is distinct from other nerve injury derived NP (possibly due to postoperative radiotherapy) and most patients may not benefit from Na^+^ -channel blockers.

### Clinical BE and sensory mapping

ALND has been associated with persistent post-surgical pain [32], which could be due to the increased risk for perioperative ICBN lesions [15]. In our cohort, however, the presence of sensory gain in BE associated with more intense pain, but not with the type of surgery or treatment. Most patients with sensory gain in BE, reported hyperesthesia/hyperalgesia to pinprick, which may involve both central and peripheral sensitization mechanisms [36].

Although sensory loss was the most common finding in both BE and QST, the individual outcomes of corresponding tests in BE and QST were consistent in only 29–57% of the patients. Similar results were observed in a previous study on 32 patients with traumatic partial nerve injury [37]. The differences in the applied stimuli and conduct of the measurement may explain this inconsistency. Since QST examines a restricted area of interest with a standardized protocol, it may reveal impairment in the sensory function that does not emerge in BE [38].

The standardized protocol, training, equipment, and reference data of the DFNS QST aim to improve quality and comparability of the data from different centers. However, although BE is more robust and mainly qualitative it is a critical step of NP diagnostics and it allows the sensory mapping of the whole affected area. BE and QST are complimentary, rather than substitutive methods of assessment of the sensory function. QST offers a means to stratify patients to certain sensory phenotypes that is not accessible by BE. This could be important to consider in the future if the sensory phenotype-based treatment design gains further support [13].

### Strengths and limitations

Strengths of the study are a relatively large and thoroughly characterized patient cohort of post-surgical NP patients. One examiner conducting BE to all patients excludes the need to consider inter-rater variability.

Most of the patients did not use regular NP medications or other analgesics. These medications may affect the sensory profile, especially the evoked pain thresholds. However, the patients reported of scarce usage of these medications.

Lack of healthy controls is a limitation of the study, since the DFNS normative data were from the back [27]. However, we were able to compare the affected and the unaffected side with each patient as her own control. Some QST items show high levels of variation among healthy individuals [26]. Therefore, a reliable set of normative values may require a large number of healthy controls. Normative data collected from multiple centers would benefit further studies of the sensory function in breast cancer patients.

## Conclusions

We describe DFNS QST sensory profiling and sensory mapping of patients with post-surgical NP several years after breast cancer treatments. DFNS QST may improve NP patient phenotyping, which cannot be achieved by BE, and lead more precise NP treatment strategies. In addition, QST may provide information on perioperative nerve injuries, which may help in post-surgical NP diagnostics. Our results suggest that post-surgical NP after breast cancer surgery differs from other nerve injury derived NP. We need further studies combining QST to specific responses to NP medications.

## References

[1] Haroutiunian S, Nikolajsen L, Finnerup NB (2013). The neuropathic component in persistent postsurgical pain: a systematic literature review. Pain.

[2] Ilhan E, Chee E, Hush J (2017). The prevalence of neuropathic pain is high after treatment for breast cancer: a systematic review. Pain.

[3] Mejdahl MK, Andersen KG, Gartner R (2013). Persistent pain and sensory disturbances after treatment for breast cancer: six year nationwide follow-up study. BMJ.

[4] Mustonen L, Aho T, Harno H (2019). What makes surgical nerve injury painful? A 4-year to 9-year follow-up of patients with intercostobrachial nerve resection in women treated for breast cancer. Pain.

[5] Pereira S, Fontes F, Sonin T (2017). Neuropathic pain after breast cancer treatment: characterization and risk factors. J Pain Symptom Manag.

[6] Warrier S, Hwang S, Koh CE (2014). Preservation or division of the intercostobrachial nerve in axillary dissection for breast cancer: meta-analysis of randomised controlled trials. Breast.

[7] Jung BF, Ahrendt GM, Oaklander AL (2003). Neuropathic pain following breast cancer surgery: proposed classification and research update. Pain.

[8] Baron R, Binder A, Wasner G (2010). Neuropathic pain: diagnosis, pathophysiological mechanisms, and treatment. Lancet Neurol.

[9] Maier C, Baron R, Tolle TR (2010). Quantitative sensory testing in the German Research Network on Neuropathic Pain (DFNS): somatosensory abnormalities in 1236 patients with different neuropathic pain syndromes. Pain.

[10] Demant DT, Lund K, Vollert J (2014). The effect of oxcarbazepine in peripheral neuropathic pain depends on pain phenotype: a randomised, double-blind, placebo-controlled phenotype-stratified study. Pain.

[11] Treede RD (2019). The role of quantitative sensory testing in the prediction of chronic pain. Pain.

[12] Cruccu G, Sommer C, Anand P (2010). EFNS guidelines on neuropathic pain assessment: revised 2009. Eur J Neurol.

[13] Baron R, Maier C, Attal N (2017). Peripheral neuropathic pain: a mechanism-related organizing principle based on sensory profiles. Pain.

[14] Vollert J, Maier C, Attal N (2017). Stratifying patients with peripheral neuropathic pain based on sensory profiles: algorithm and sample size recommendations. Pain.

[15] Andersen KG, Duriaud HM, Kehlet H (2017). The relationship between sensory loss and persistent pain 1 year after breast cancer surgery. J Pain.

[16] Gottrup H, Andersen J, Arendt-Nielsen L (2000). Psychophysical examination in patients with post-mastectomy pain. Pain.

[17] Schreiber KL, Martel MO, Shnol H (2013). Persistent pain in postmastectomy patients: comparison of psychophysical, medical, surgical, and psychosocial characteristics between patients with and without pain. Pain.

[18] Demant DT, Lund K, Finnerup NB (2015). Pain relief with lidocaine 5% patch in localized peripheral neuropathic pain in relation to pain phenotype: a randomised, double-blind, and placebo-controlled, phenotype panel study. Pain.

[19] Vollert J, Attal N, Baron R (2016). Quantitative sensory testing using DFNS protocol in Europe: an evaluation of heterogeneity across multiple centers in patients with peripheral neuropathic pain and healthy subjects. Pain.

[20] Meyer-Frießem CH, Attal N, Baron R (2020). Pain thresholds and intensities of CRPS type I and neuropathic pain in respect to sex. Eur J Pain.

[21] Dezawa K, Noma N, Watanabe K (2016). Short-term effects of orthognathic surgery on somatosensory function and recovery pattern in the early postoperative period. J Oral Sci.

[22] Kaunisto MA, Jokela R, Tallgren M (2013). Pain in 1,000 women treated for breast cancer: a prospective study of pain sensitivity and postoperative pain. Anesthesiology.

[23] Finnerup NB, Haroutounian S, Kamerman P (2016). Neuropathic pain: an updated grading system for research and clinical practice. Pain.

[24] Andersen KG, Aasvang EK, Kroman N (2014). Intercostobrachial nerve handling and pain after axillary lymph node dissection for breast cancer. Acta Anaesthesiol Scand.

[25] Cleeland CS, Ryan KM (1994). Pain assessment: global use of the Brief Pain Inventory. Ann Acad Med Singapore.

[26] Rolke R, Baron R, Maier C (2006). Quantitative sensory testing in the German Research Network on Neuropathic Pain (DFNS): standardized protocol and reference values. Pain.

[27] Pfau DB, Krumova EK, Treede RD (2014). Quantitative sensory testing in the German Research Network on Neuropathic Pain (DFNS): reference data for the trunk and application in patients with chronic postherpetic neuralgia. Pain.

[28] Vollert J, Magerl W, Baron R (2018). Pathophysiological mechanisms of neuropathic pain: comparison of sensory phenotypes in patients and human surrogate pain models. Pain.

[29] Vilholm OJ, Cold S, Rasmussen L (2009). Sensory function and pain in a population of patients treated for breast cancer. Acta Anaesthesiol Scand.

[30] Schuning J, Scherens A, Haussleiter IS (2009). Sensory changes and loss of intraepidermal nerve fibers in painful unilateral nerve injury. Clin J Pain.

[31] Fernandez-Lao C, Cantarero-Villanueva I, Fernandez-de-las-Penas C (2011). Widespread mechanical pain hypersensitivity as a sign of central sensitization after breast cancer surgery: comparison between mastectomy and lumpectomy. Pain Med.

[32] Andersen KG, Duriaud HM, Jensen HE (2015). Predictive factors for the development of persistent pain after breast cancer surgery. Pain.

[33] Simpson DM, Schifitto G, Clifford DB (2010). Pregabalin for painful HIV neuropathy: a randomized, double-blind, placebo-controlled trial. Neurology.

[34] Campbell JN, Meyer RA (2006). Mechanisms of neuropathic pain. Neuron.

[35] La Cesa S, Sammartino P, Mollica C (2018). A longitudinal study of painless and painful intercostobrachial neuropathy after breast cancer surgery. Neurol Sci.

[36] Jensen TS, Finnerup NB (2014). Allodynia and hyperalgesia in neuropathic pain: clinical manifestations and mechanisms. Lancet Neurol.

[37] Leffler AS, Hansson P (2008). Painful traumatic peripheral partial nerve injury-sensory dysfunction profiles comparing outcomes of bedside examination and quantitative sensory testing. Eur J Pain.

[38] Teerijoki-Oksa T, Forssell H, Jaaskelainen SK (2019). Validation of diagnostic methods for traumatic sensory neuropathy and neuropathic pain. Muscle Nerve.

